# Exploiting Extracellular Vesicles Strategies to Modulate Cell Death and Inflammation in COVID-19

**DOI:** 10.3389/fphar.2022.877422

**Published:** 2022-05-20

**Authors:** Barbara Bortot, Arianna Romani, Giuseppe Ricci, Stefania Biffi

**Affiliations:** ^1^ Institute for Maternal and Child Health, IRCCS Burlo Garofolo, Trieste, Italy; ^2^ Department of Translational Medicine and LTTA Centre, University of Ferrara, Ferrara, Italy; ^3^ Department of Medicine, Surgery and Health Sciences, University of Trieste, Trieste, Italy

**Keywords:** COVID-19, extracellular vesicles, inflammation, cell death, inhibitors of MDM2, p53, drug delivery

## Abstract

The coronavirus disease (COVID-19) is responsible for more than 5 million deaths worldwide, with respiratory failure being the most common clinical presentation. COVID-19 complications still present a considerable burden on healthcare systems, and signs of the post-COVID syndrome are concerns for potential long-term damages. An increasing body of evidence highlights extracellular vesicles’ (EVs) relevance in modulating inflammation and cell death in the diseases related to these processes. Several types of EVs-based investigational new drugs against COVID-19 have been approved by the US Food and Drug Administration to initiate a Phase I/II trial under an Investigational New Drug protocol. EVs can be employed as natural drug delivery nanoparticle-based systems due to their inherent potential in transferring material between cells, their natural origin, and their capability to encapsulate various biological molecules, offering an exciting alternative for administering drugs acting on the cell cycle control. In this context, small-molecule inhibitors of Mouse Double Minute 2 (MDM2) such as Nutlin-3 and Idasanutlin by promoting p53 survival and its antiviral activity might be helpful to modulate the IFN signalling pathway and reduce the overall pro-inflammatory burden.

## Introduction

The World Health Organization declared the novel coronavirus (COVID-19) outbreak a global pandemic on 11 March 2020 (Cucinotta and Vanelli, 2020). COVID-19, caused by the severe acute respiratory syndrome-coronavirus-2 (SARS-CoV-2), has become a significant public health problem worldwide, with extremely high morbidity and mortality rates (Cascella et al., 2021). The primary receptor for host cell attachment and subsequent host cell entry for the SARS-CoV-2 virus is the angiotensin-converting enzyme II (ACE2) (Zhang et al., 2021). The broad expression of ACE2 in many tissues in humans contributes to the multiple tissues infection by SARS-CoV-2, which can affect major organ systems such as the gastrointestinal tract, hepatobiliary, cardiovascular, renal, and central nervous system (Cascella et al., 2021). However, since the ACE2 receptor is highly expressed on alveolar type-II epithelial cells and ciliated cells in the lungs, SARS-CoV-2 mainly leads to pneumonia. Most patients infected with SARS-CoV-2 exhibit mild-to-moderate respiratory infection symptoms, but sudden clinical worsening into acute respiratory distress syndrome (ARDS) leads to intubation and mechanical ventilation in some patients. In the pathogenesis of ARDS due to COVID-19, the prominent role is played by an uncontrolled immune response with rapidly developing severe life-threatening excessive production of proinflammatory cytokines (refer to as cytokine storm) (Shimizu, 2019; Ragab et al., 2020). Continuous activation and expansion of immune cells, lymphocytes, and macrophages produce a massive amount of cytokines, resulting in a cytokine storm, whose clinical findings are attributed mainly to the action of the proinflammatory cytokines like IL-1, IL-6, IL-18, IFN-*γ*, and TNF-*α*. Cytokine release syndrome threatens the emergence and progression of ARDS.

A comprehensive characterization of nature and the extracellular vesicles (EVs) components released in a pathological context is needed to understand how metabolic alterations affect the disease microenvironment and therapeutic response; we have reported our experience in ovarian cancer research (Bortot et al., 2021). According to the available literature, EVs can regulate inflammation and regenerative responses by modulating anti-inflammatory cytokine concentration and switching the immune cell to a regenerative secretome. The exciting roles of EVs in virus infection, immune response, and inflammation provide a new perspective on the treatment of COVID-19 (Dogrammatzis et al., 2020). As mediators of intercellular communication, EVs can modulate gene expression and cellular pathways in recipient cells, and they can subsequently induce signalling effects that can be exploited therapeutically. Viral infection may change EVs’ protein or RNA expression profile in the infected cells, leading to a modulation of the host’s immune response (Hassanpour et al., 2020).

Experimental studies have demonstrated that mesenchymal stem cells (MSCs) can effectively treat lung inflammation and pathological lung tissue damage caused by various lung injuries (Jamshidi et al., 2021; Schultz et al., 2021). Many studies associate the anti-inflammatory effect of MSCs with their secretome, which includes a large number of soluble factors and EVs. MSC-derived exosomes are believed to have the same therapeutic effect on pneumonia as MSCs themselves and an actual regenerative stimulating result on several wounds. In particular, inhalation of MSC-EVs may reduce inflammation and damage to the lung tissue and stimulate the regenerative processes. MSCs-EVs have a safer profile than MSCs, whose intravenous administration can determine aggregation phenomena at the level of the injured microcirculation and the intrinsic risk of mutagenicity and oncogenicity (Maumus et al., 2020). Moreover, MSCs-EVs over MSCs can be stored for long terms, allowing safe transportation and storage for therapeutic use (Maumus et al., 2020).

## Supporting Evidence

### Immune Reaction in COVID-19: Innate Immunity and the Role of P53

**FIGURE 1:**
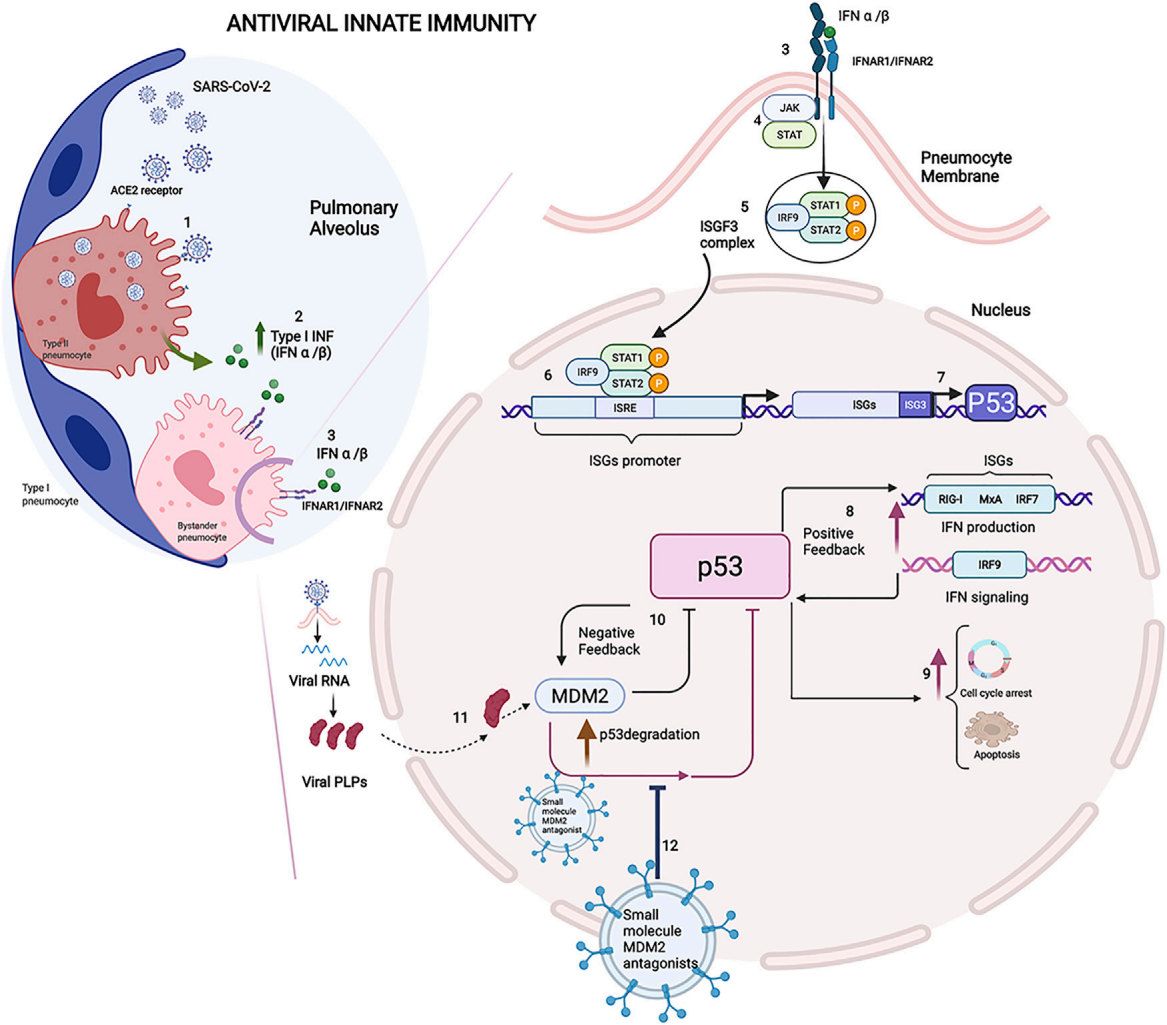
Immune reaction in COVID-19: Innate immunity and the role of P53. **1**) SARS-CoV-2 infects the type II pneumocytes by binding its S protein with the angiotensin-converting enzyme 2 (ACE2) receptor exposed on the surface of the pneumocyte membrane. **2**) IFN*α* and IFN*β* play an antiviral role inside the infected cells by inhibiting viral replication. **3**) On the neighbour cells, IFN*α* and IFN*β* bind their receptors IFNAR1 and IFNAR2. **4**) The signal transduction results in activation of the JAK-STAT signalling pathway involved in IFN-dependent antiviral defence. **5**) The phosphorylated STAT1/STAT2 proteins form a heterotrimeric transcriptional factor with IRF9 known as the IFN-stimulated gene factor 3 (ISGF3), which translocates to the nucleus. **6**) ISGF3 ties the IFN sequence response elements (ISREs) contained within the IFN-stimulated genes (ISGs) promoter, and it enhances the transcription of the downstream genes involved in IFN antiviral response (Platanias and Fish, 1999). **7)** ISREs is included in the P53 gene promoter, and ISGF3 was proved to induce the expression of P53, whose protein level increases during the antiviral immune response. **8**) P53 protein actives IFN production and signalling modulating the pathway members, such as IRF9, with positive feedback in the early stages of infection. **9**) In the later stage of infection, p53 could thwart the viral spread leading to apoptosis in infected cells (Muñoz-Fontela et al., 2008). **10**) The level of p53 is controlled by E3 ligase murine double minute 2 (MDM2), and the MDM2-p53 interaction decreases the p53 transcription factor and downregulates mdm-2 gene expression creating a negative feedback mechanism (Wu et al., 1993). **11**) Viral papain-like proteases (PLPs), a cysteine protease essential to process the viral proteins, suppresses the innate immunity by stabilizing the cellular oncoprotein MDM2 and inducing the degradation of p53. **12**) The therapeutic strategy consists of the administration of the extracellular vesicles loaded with small-molecule MDM2 inhibitors to block the protein-protein interaction between p53 and MDM2. Their ability to protect p53 from degradation makes the small molecules MDM2 inhibitors an attractive drug that, once loaded in the EVs, could be delivered to the targeted district, reducing the pro-inflammation state in the early stages of viral infection. The Figure was created with Biorender.com.

An increasing body of evidence indicates that SARS-CoV-2 enters the epithelial cells of the respiratory tract and lungs and epithelial and nonepithelial cells of other organs that express the ACE2 receptor, which triggers an antiviral immune response upon detection of the virus (Sungnak et al., 2020; Zhang et al., 2022). SARS-CoV-2 infects the type II pneumocytes by binding its S protein with the angiotensin-converting enzyme 2 (ACE2) receptor exposed on the surface of the pneumocyte membrane. The entry of SARS-CoV-2 is facilitated by the transmembrane serine protease 2 (TMPRSS2), and viral RNA is released within virus-induced double-membrane vesicles (DMVs) in the cytoplasm of the infected cells (Gurunathan et al., 2021). The SARS-CoV-2 RNA present in the cytoplasm is a ligand of the retinoic acid-inducible gene I (RIG-I)- like receptors (RLRs), and their binding leads to a cascade of reactions which triggers the innate interferon (IFN) response *via* the activation of type I IFN *α* and IFN *β* gene expression (Xia et al., 2020). IFN*α* and IFN*β* play an antiviral role inside the infected cells by inhibiting viral replication. Subsequently, after being released into the pulmonary alveolus environment by infected cells, these cytokines start an antiviral state by acting as the main effectors of the host immune response (Figure 1). Of note, p53 is activated in virally infected cells to evoke an apoptotic response, and it is critical for the antiviral defence of the host (Takaoka et al., 2003). In particular, at the early phase in the infection cycle, virus-infected pneumocytes produce IFN-*α*/*β* and eventually undergo p53-dependent apoptosis. On the other hand, virus-induced IFN-*α*/*β* may act on the surrounding, uninfected cells to support antiviral responses by inducing cellular genes that inhibit virus replication and, in addition, by increasing p53 activation to promote apoptotic cell fate (Figure 1).

Further research is warranted to clarify how SARS-CoV-2 modulates the type-I IFN response early during infection. A recent study revealed that SARS-CoV-2 induced an aberrant delayed type-I IFN response in cultured cells, scarcely induced early during viral infection while raised at late time points. This delayed antiviral response may be critical for virus replication. Indeed, a significant activity of viral transcription was observed before the IFN induction in SARS-CoV-2-infected cells (Lei et al., 2020). Moreover, SARS-CoV-2 is sensitive to IFN pretreatment, suggesting that IFN therapy could be an option for COVID-19 treatment (Lei et al., 2020; Mantlo et al., 2020). A prospective observational study demonstrated that serum IFN-I levels in the early phase of SARS-CoV-2 infection were higher in patients who developed hypoxemic respiratory failure and could be a predictor of disease progression, including respiratory failure (Nagaoka et al., 2022).

Coronaviruses have evolved several strategies to evade the host immune defence targeting the IFN production and signalling pathways and increasing IFN resistance (Xia et al., 2020). The mechanism used to inhibit the IFN signalling pathway targets p53 and its antiviral activity. In particular, papain-like proteases (PLPs) of coronaviruses have a cysteine protease activity to process the viral proteins and suppress innate immunity by deubiquitinating and stabilizing the cellular oncoprotein MDM2 and inducing the degradation of p53 (Yuan et al., 2015).

In severe COVID-19, a high virus load hyperactivates the innate immune system, resulting in high levels of inflammatory cytokines, a condition that has been defined as “cytokine storm” (Schultze and Aschenbrenner, 2021). However, recent findings support a more complex immune response, which could be better summarized as viral sepsis, characterized by T cell deficiencies, systemic hyper inflammation, and COVID-19-associated coagulopathy (Riva et al., 2020).

### MDM2 Inhibitors

Acute respiratory distress syndrome (ARDS), which emerge in COVID-19 patients, is caused by dysfunctional pulmonary endothelium sustained by inflammatory processes, which *in vitro* can be mimicked by LPS induction. LPS treatment suppresses P53 protein expression (Barabutis et al., 2015) on human lung microvascular endothelial cells; thus, p53 activators such as MDM2 inhibitors in coronavirus infected cells might be effective drug targets in therapy against the COVID-19 virus (Ramaiah, 2020).

The small molecules MDM2 inhibitors represent a new class of selective antagonist agents developed to block the protein-protein interaction between p53 and MDM2 (Beloglazkina et al., 2020; Zhao et al., 2015). These molecules are small non-peptide compounds, and their design imitates the *α*-helical p53 peptide structure binds to MDM2 (Zauli et al., 2020). Nutlins (-1,-2,-3) were the first class of selective and potent MDM2 inhibitors developed. They were designed to mimic the *α*-helical P53 structure interfering with P53-MDM2 binding. Nutiln-3a is the most potent compound of the family, which has been proposed as a valuable candidate in restraining cytokine storm and cells death after coronavirus infection (Ramaiah, 2020; Zauli et al., 2020). Indeed, *in vitro* and *in vivo*, Nutin-3a stimulates cell cycle arrest, senescence and apoptosis-associated to P53 stabilization (Secchiero et al., 2008). Moreover, it has been reported that Nutlin-3a reduces the inflammatory factors (IL-1*α*, IL6) produced by cells triggered by genotoxic stimuli (Wiley et al., 2018). These data align with a recent study on the mice model where LPS treatment induced a more robust inflammatory response in P53 KO mice than their littermates (Uddin et al., 2020), suggesting a P53 protective role on inflammatory lung injury.

### Extracellular Vesicles as Efficient Drug Carriers

Drug delivery systems based on engineered nanomaterials offer a versatile therapeutical platform with excellent loading capacity, high biocompatibility, and tunable pharmacokinetics. However, despite consistent results concerning drug toxicity reduction in many biomedical studies, their use has not always shown improvements in clinical outcomes, measured by response rate and survival, and there are only a few nano-drug systems in the clinical setting (Biffi et al., 2019; Fu et al., 2021). EVs can be employed as natural drug delivery systems due to their inherent potential in transferring material between cells, their low immunogenity, and their capability to encapsulate various biological molecules, offering an exciting alternative to the engineered nanomaterials (Fu et al., 2021). The biogenesis of EVs has not been fully elucidated, but commonly recognized pathways produce three vesicle types, including 1) exosomes (30–200 nm); 2) microvesicles (200–2000 nm); and 3) apoptotic bodies (>1,000 nm). Among all subtypes of EVs, exosomes that exhibit a comparable size range to engineered nanoparticles have been applied as drug delivery vehicles giving hopes for future clinical applications (Fu et al., 2021). Compared to artificial drug delivery systems (e.g., liposomes, polymers) with up to 90% of the administrated dose captured by the liver, being a primary barrier that hampers the nanoparticles from potential clinical therapeutics, EVs could minimize their clearance by the mononuclear phagocyte system, prolonging the *in vivo* circulation time and decreasing the distribution to the liver and spleen (Belhadj et al., 2020). The autophagic-lysosomal pathway constitutes a further barrier, which results in intracellular degradation of internalized drugs. Contrary to engineered nanoparticles, EVs are endowed with unique biochemical composition in their membrane rich in sphingomyelin, cholesterol and de-saturated lipids, enabling them to resist lysosomal degradation, thus improving the number of cargos being unleashed into the cytoplasm (Skotland et al., 2017; Fu et al., 2021).

EVs’ mechanical properties play essential roles in critical aspects of drug delivery, such as uptake by cells and transport through tissues. Hence, similar to engineered nanoparticles whose mechanical properties are determined by several physicochemical features (e.g., particle size, shape, surface composition), natural EVs can be modified to modulate their mechanical properties and, thus, their delivery performance (Kutvonen et al., 2012; Fu et al., 2021).

Despite their many advantages, a significant limitation that hinders pharmaceutical EVs’ clinical translation is their low-efficient bioproduction (Yang et al., 2020).

**FIGURE 2:**
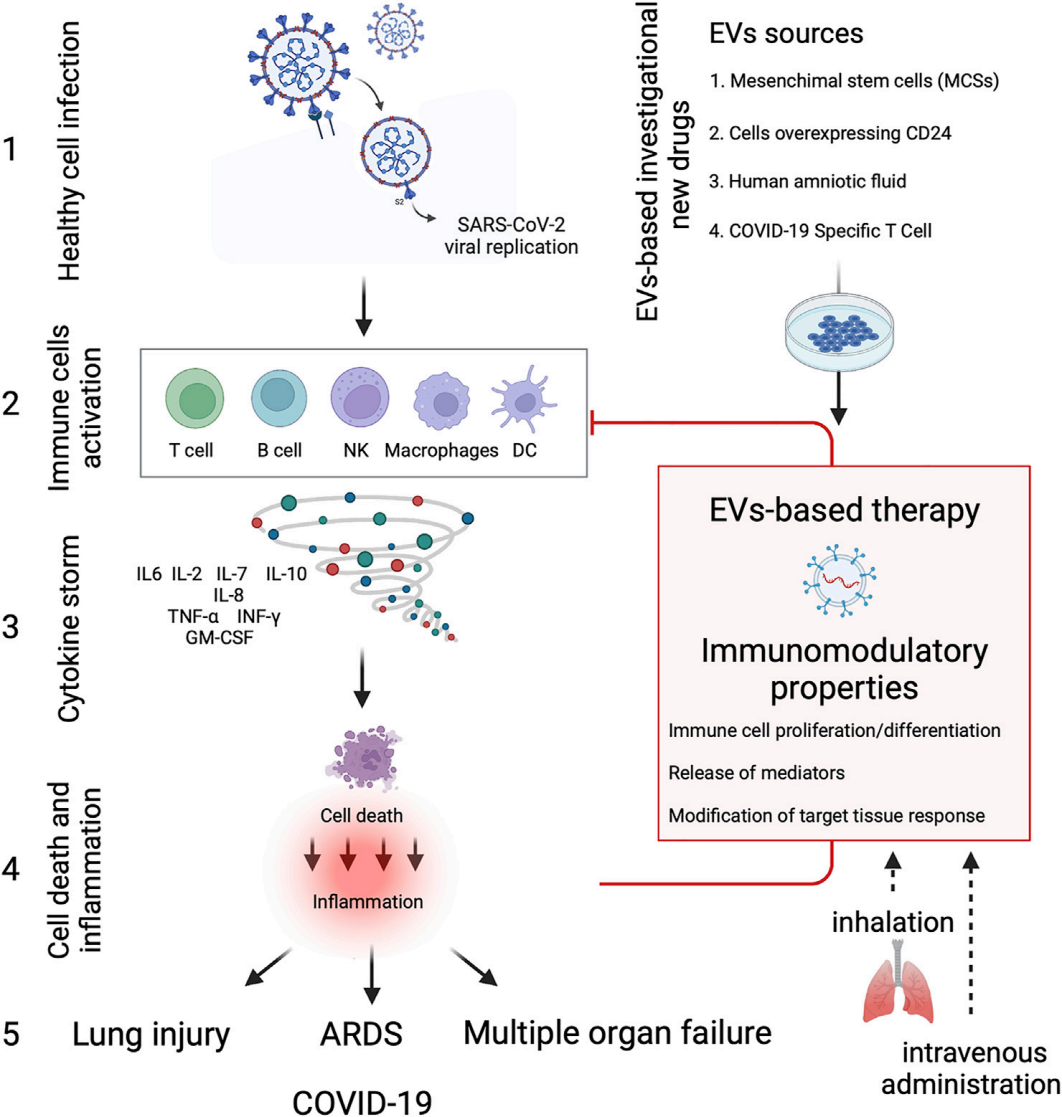
EVs-based therapy in COVID-19. Diagram showing the immune cells activation and cytokine storm produced due to SARS-CoV-2 infection, leading to severe inflammation and tissue dysfunction. EVs-based investigational new drugs against COVID-19 can be classified as **1)** EVs derived from allogeneic mesenchymal stem cells (MSCs), **2)** EVs overexpressing CD24 isolated and purified from human embryonic kidney T-REx™-293 cells engineered to express high levels of human CD24; **3)** EVs extract from human amniotic fluids (Zofin) and **4)** EVs derived from allogenic COVID-19 T cells. EVs-based therapies possess immunomodulatory properties and can suppress and inhibit innate and adaptive immune cells’ activation, maturation and proliferation, downregulate cytokine storm, and modify the target tissue response. Besides the conventional intravenous administration, EVs-based therapies can reduce the inflammatory response in the lung through inhalation, thus regenerating the damaged alveolar epithelium and endothelium at a lower concentration dose. The Figure was created with Biorender.com.

Clinical EVs-based studies are on the rise, and we have found 14 clinical trials listed on ClinicalTrials.gov with “exosomes AND COVID-19” or “extracellular vesicles AND COVID-19” as search terms as of December 2021 (Table 1). Several types of EVs-based investigational new drugs against COVID-19 have been approved by the US Food and Drug Administration (FDA) to initiate a Phase I/II trial under an Investigational New Drug (IND) protocol, and they can be classified as 1) EVs derived from allogeneic MSCs; 2) EVs overexpressing CD24 isolated and purified from human embryonic kidney T-Rex™-293 cells engineered to express high levels of human CD24; 3) EVs extract from human amniotic fluids (Zofin) and 4) EVs derived from Allogeneic COVID-19 T cells (Figure 2). EVs-based therapies possess immunomodulatory properties and can suppress innate and adaptive immune cells’ activation, maturation and proliferation, downregulate cytokine storm, and modify the target tissue response.

**TABLE 1:** EVs-based investigational new drugs against COVID-19 approved by the US Food and Drug Administration (FDA) to initiate a Phase I/II trial under an Investigational New Drug (IND) protocol.

ClinicalTrials.gov identifier (Phase)	Source of EVs
NCT04276987 (Phase I)	Allogenic adipose mesenchymal stem cells (MSCs)
NCT04384445 (Phase I and II)	Biological extract from human amniotic fluids (Zofin)
NCT04491240 (Phase I and II)	MSCs
NCT04602442 (Phase II)	MSCs
NCT04798716 (Phase I and II)	MSCs
NCT04747574	EVs overexpressing CD24 isolated and purified from human embryonic kidney T-REx™-293 cells engineered to express high levels of human CD24
NCT04389385 (Phase I)	Allogenic COVID-19 T cells
NCT04657406 (Phase II)	Biological extract from human amniotic fluids (Zofin)
NCT04493242 (Phase II)	Allogenic bone marrow MSCs
NCT05125562	Allogenic bone marrow MSCs
NCT04657458	Allogenic bone marrow MSCs
NCT05116761	Allogenic bone marrow MSCs
NCT04969172	EVs overexpressing CD24 isolated and purified from human embryonic kidney T-REx™-293 cells engineered to express high levels of human CD24
NCT04902183	EVs overexpressing CD24 isolated and purified from human embryonic kidney T-REx™-293 cells engineered to express high levels of human CD24

## The Potential Role of Small-Molecule Inhibitors of MDM2 in the Treatment of COVID-19 Disease

SARS-CoV-2 could affect the dynamic equilibrium between p53 and MDM2 and the use of MDM2 inhibitors could limit the degradation of p53, reducing the pro-inflammation state (Zauli et al., 2020) (Figure 1).

It is noteworthy that a group of SARS-CoV2 accessory proteins (e.g., ORF3a, ORF3b, ORF6, ORF7a, ORF8, ORF9b, and ORF9c) act as IFN-I antagonists, thus contributing to the disease pathogenesis and exacerbating the virulence (Redondo et al., 2021). It has been demonstrated *in vitro* that ORF9b, in particular, can suppress the type I and III IFNs production by affecting multiple molecules involved in the innate antiviral immunity pathways. Also, ORF9b interacts with Tom70 protein, a mitochondrial import receptor, and this complex may control the host immune response by affecting type I IFN synthesis (Redondo et al., 2021).

The therapeutic strategy here discussed considers the broad diversity in p53 controls and functions. The basal p53 levels are sufficient to drive the ISG gene expression, including IRF9 and IRF7 transcriptional factor genes involved in the IFN production and signalling pathways to establish the IFN-dependent antiviral state. Experimental pieces of evidence support that *in vivo* p53 protein contributes to enhancing IFN signalling and IFN antiviral immunity and indicate that the p53 role in inducing apoptosis is not essential for antiviral functions during the initial period after *in vivo* infection (Muñoz-Fontela et al., 2008). Therefore, as SARS-CoV2 accessory proteins’ action hinders the IFN-I production, MDM2 inhibitors could be helpful by lowering the p53 protein ubiquitination in the cytoplasm and allowing the up-regulation of IFN-dependent antiviral state. In later stages, p53 prompting apoptosis could eliminate the infected cells thwarting the viral replication.

Due to its poor bioavailability *in vivo*, to date, the use of Nutlins was limited to preclinical study; nevertheless, second-generation molecules like Idasanutlin (RG7388) that possess the identical cellular mechanism with enhanced potency, selectivity and bioavailability compared to first-generation ones are used in clinical studies (Konopleva et al., 2020). Idasanutlin could be orally administrated and has been demonstrated to have 1 day of half-life and a dose-dependent stabilization of p53 protein (Khurana and Shafer, 2019; Pápai et al., 2019). From phase I/Ib clinical study on acute myeloid leukaemia, Idasanutlin has been proposed as monotherapy and combination therapy with antileukemic drugs (Montesinos et al., 2020).

## Discussion

At the end of 2021, several countries approved vaccines for COVID-19 through their respective regulatory agencies, but no specific treatment has yet been officially acknowledged to treat COVID-19. Even though COVID-19 mRNA vaccines have shown efficacy in preventing severe disease (Thompson et al., 2021), recent reports suggest that SARS-CoV-2 variants may promote immune escape mechanisms or annihilate the immune response induced by existing vaccines (Planas et al., 2021). Moreover, COVID-19 complications still present a considerable burden on healthcare systems. Many patients develop a post-COVID-19 syndrome characterized by a wide range of chronic symptoms for weeks or months after infection, often neurological, cognitive or psychiatric (Carod-Artal, 2021). A significant percentage of survivors of severe COVID-19 present fibrotic-like radiographic abnormalities long after hospitalization, which is concerning for potential long-term damages (McGroder et al., 2021).

Many studies are conducted to test different therapeutic approaches in this context, and the FDA is currently exploring various single-agent and combination treatments for the COVID-19 disease. Mechanistically, therapeutic agents can be classified in 1) targeting the viral life cycle, such as inhibitors of the viral RNA polymerase (e.g., remdesivir) or protease inhibitors (e.g., lopinavir-ritonavir), in 2) SARS-CoV-2–targeted antibody therapies, and 3) drugs focused on the host response, such as immunomodulators, glucocorticoids.

It should be emphasized that the fundamental cause of the most severe forms of COVID-19 disease is immediately evident: the immune system overactivation and the cytokine storm that often ensues. EV-based therapies, mainly the MSCs-derived EVs, are intensively studied to treat COVID-19 due to their immunomodulation capacity (Figure 2). These formulations are composed of different vesicles and a variable portion of soluble proteins/extracellular matrix components involved in the final product’s biological activity. Therefore, the most relevant term to define them would ultimately be “EV-enriched secretome” rather than “EVs.” Components of the EV-enriched secretome may interact with the target cells through ligand-receptor binding or by internalization to modulate cellular responses. Moreover, several paracrine factors can interact directly with immune cells, including T cells, B cells, dendritic cells, macrophages, and natural killer cells, thus inhibiting the over activation of the immune system and preventing the cytokine storm induced by the SARS-Cov2 infection. EV-based therapy has significant advantages over cell-based and monoclonal antibody treatments due to its low immunogenicity and tumorigenicity, easier manipulation, less manufacturing time and lower cost.

EVs can be employed as natural drug delivery nanoparticle-based systems due to their inherent potential in transferring material between cells, their natural origin, and their capability to encapsulate various biological molecules. Drug delivery relies on the formulation of the treatment to reach its target site and achieve therapeutic efficacy with optimized toxicity and safety. The drug delivery concept with innovative nanoparticle-based systems has gained considerable interest and offer applicative potential in the medical field (Biffi et al., 2015). In particular, a nanoparticle-based system allows to store large drug payloads within the NP core, and the possibility of engineering the nanoparticle with surface “tissue-specific targeting” molecules allows a specific drug delivery and an improved targeting/localization, which result in greater efficacy, tissue specificity and reduced side effects (Biffi et al., 2015; Biffi et al., 2019). EVs have already shown potential in cancer active targeting and immunotherapy and have been recently recognized by several authors as the most promising drug delivery system (Biffi et al., 2019). Several EVs have demonstrated some efficacy in this regard concerning agents that can improve delivery and deposition in the small airways and alveolar regions *via* the modality of inhalation therapy (Kadota et al., 2022). Since the route of infection and disease progression is primarily through the lungs, the drug delivery directly to the lungs upon inhalation is the most appropriate route of administration for treating COVID-19. Noteworthy, the International Society for Aerosols in Medicine (ISAM) has called for the development of inhaled therapies for COVID-19 treatment, as the inhaled doses of therapy candidates in development required to achieve high lung concentrations are typically much lower than their therapeutic doses by other routes (Mitchell et al., 2020). However, the precise dosing strategy will depend on pulmonary pharmacokinetics. As detailed in the new U.S. Food and Drug Administration guidance (Center for Drug Evaluation and Research and Center for Biologics Evaluation and Research, 2020), Phase 1a clinical studies will still be required to establish pulmonary safety before evaluating the inhaled therapy’s primary efficacy. However, to decrease the extent and duration of Phase 1a studies and more quickly enable dosing in a Phase 1b trial to evaluate dose and effectiveness, existing and *in silico* based pharmacokinetic modelling data could be leveraged to support clinical studies.

Treatment based on MDM2 inhibitors delivered by EVs should be considered for further evaluation for COVID-19 patients. Previous reports indicate enhanced efficacy of Nutnin-3a loaded into poly (lactide-co-glycolide) (PLGA) nanoparticles (NP) against haematological cancers. Nanoparticles incorporated with Nutlin-3a reduced the subcutaneous tumour volume and promoted induction of apoptosis in a xenograft mouse model (Voltan et al., 2013). In addition, recent clinical studies indicate that a second-generation potent and selective MDM2 antagonist (i.e., Idasanutlin) can have a markedly beneficial impact on p53 activation in leukemic patients, yet along with common adverse side effects such as diarrhoea, nausea/vomiting and, in some cases, myelosuppression causing febrile neutropenia and thrombocytopenia, possibly caused by an on-target impact on normal cells (Konopleva et al., 2020).

Promoting p53 activation by targeting its inhibitor (MDM2) using a nanoparticle-based system potentially offers the chance to increase the efficacy in harming the infected cells limiting the inflammatory burden, modulating the IFN signalling pathway and promoting apoptosis. We envisage the early phase of the infection cycle as the optimal time window for MDM2 inhibitors administration. However, the clinically observed disease course shows enormous heterogeneity, and the induction of p53 by inhibiting MDM2 might trigger different magnitude of cellular responses. During a health emergency context, as in the case of the COVID-19 pandemic, therapeutic approaches are often allowed to prevent the disease impact. However, after an early observational study, it is crucial to conduct randomized, double-blinded, placebo-control trials to fully ascertain the efficacy of new investigational drugs.

Furthermore, it is indispensable to clarify the best time window for administration and underlying molecular mechanisms through which new investigational drugs benefit patients. Therefore, future studies focusing on the impact of modulating cell death and inflammation pathways would lead research further in the right direction, and preclinical and clinical investigation with mechanistic approaches are highly required. Future experiments will be crucial to reveal the EV-mediated crosstalk in lung repair and regeneration, facilitating the development of novel EV therapeutics.

## Data Availability

The original contributions presented in the study are included in the article, further inquiries can be directed to the corresponding author.
